# ABO and Rhesus (D) blood group distribution among blood donors in rural south western Uganda: a retrospective study

**DOI:** 10.1186/s13104-016-2299-5

**Published:** 2016-12-21

**Authors:** Richard Onyuthi Apecu, Edgar M. Mulogo, Fred Bagenda, Andrew Byamungu

**Affiliations:** 1 Department of Medical Laboratory Sciences, Mbarara University of Science and Technology, P.O. Box 1410 Mbarara, Uganda; 2 Department of Community Health, Mbarara University of Science and Technology, P.O. Box 1410 Mbarara, Uganda; 3 Mbarara Regional Blood Bank, Southwestern Region, Ministry of Health, Mbarara, Uganda

**Keywords:** ABO, Rhesus (D), Blood groups, Rural southwestern Uganda

## Abstract

**Background:**

In Uganda, geographical distribution of blood groups and Rhesus (D) factor varies across the country. The aim of this study was to examine the distribution of these groups among voluntary blood donors in rural southwestern Uganda.

**Results:**

Twenty-three thousand five hundred four (23,504) blood donors were included in the study. The donors had a mean age of 21 years (SD ± 5.7) and were mainly male (73%). The distribution of ABO blood group was; blood group O (50.3%); blood group A (24.6%); blood group B (20.7%) and blood group AB (4.5%). The proportions of Rhesus (D) positive and Rhesus (D) negative were 98 and 2% respectively. The proportion of non-adult donors (<18 years) was significantly higher among the female than the male donors (p value <0.001). A significantly higher proportion of males than females were Rhesus (D) negative (p-value <0.001). No significant relationship was found between age and blood group distribution.

**Conclusion:**

The sequence of ABO distribution among the rural population in southwestern Uganda is; O > A > B > AB, with males as the predominant donors. The frequency of Rhesus (D) negative is very low in rural southwestern Ugandan and is mainly among males. The blood bank services in southwestern Uganda need to develop innovative strategies targeting female donors who are more likely to boost blood stocks in the region.

## Background

The discovery of the ABO blood groups by Austrian scientist Karl Landsteiner in 1900 was the greatest achievement in the history of blood transfusion medicine. He found three different blood types and he described them as A, B, and O blood groups. Alfred Von Decastello and Adrian Sturli discovered the fourth type AB in 1902. [1–3]. Forty years later, both Landsteiner and Weiner discovered Rhesus (D) antigen [4, 5, 28]. The Landsteiner’s discovery was a breakthrough in the history of blood transfusion medicine, as it opened the door to the birth of a wide spectrum of discoveries in the field of Immunoheamatology.

To date about 700 red cell antigens have been recognized by International Society of Blood Transfusion [6]. These antigens are organized into 30 human blood group systems and each person has a unique spectrum of blood groups with the exception of identical twins or triplets whose blood groups are exactly the same [7, 8]. The most important human blood group systems for blood transfusion or transplantation are the ABO and Rhesus blood systems. Red blood cells contain a series of glycoproteins and glycolipids on their surface which constitutes the blood group antigens. The bombardment of the red blood cells with A and or B antigen occurs as a consequence of the action of glycosyltransferase enzymes that add specific sugars of conformation dependent epitopes along with the Rh (D) protein from D antigen. The production of these antigens is genetically controlled.

There are many blood group systems on the basis of different blood group antigens but only ABO and Rhesus system are important in clinical practice. ABO system consists of four main groups A, AB, B, and O which are determined on the basis of presence or absence of A and B antigens. These antigens are under the control of three allelic genes, A, B and O, situated on the long arm of chromosome 9q [9]. In Rhesus (D) system, blood groups are Rh-positive or Rh-negative on the basis of presence or absence of Rhesus D antigens on red cell surface. The Rhesus antigens are determined by three pairs of closely linked allelic genes located on chromosome one [2].

All human populations share the same ABO and Rhesus blood group systems; although they differ in the frequencies and distributions of specific types in different races, ethnic groups, and socio-economic groups or amongst different populations [10, 11]. There is a paucity of literature on the frequency and distribution of the ABO blood group and Rhesus (D) blood group among the population living in southwestern Uganda. The knowledge of distribution of ABO and Rhesus (Rh) blood group is essential for effective management of blood bank inventory [12] at the regional and national levels in the case of Uganda. This study reports the distribution of ABO and Rhesus blood groups among voluntary blood donors in southwestern Uganda.

## Methods

### Design and sample population

A retrospective study was conducted using 1 year data (January 2014–December 2014) from a regional blood bank in rural southwestern Uganda. Records of 25,504 voluntary blood donors were reviewed. The donors were recruited from the nine districts in this region (Bushenyi, Ibanda, Isingiro, Kabale Kiruhura, Mbarara, Ntungamo, Rukungiri and Shema), that have a total population of 3,334,260 [13].

Prior to donating blood the donors were first assessed for physical and health wellbeing. The assessment criteria required that the donors were: body weight >45 kg; heamoglobin levels, male 13.5–17.0 g/dl and female 12.5–16 g/dl and a blood pressure of up to 160/90 mmHg were accepted. Only donors who satisfied these criteria were recruited.

Blood grouping ABO and Rhesus was done by antigen antibody micro-agglutination test using commercially available standard antisera validated at National Blood Bank. Both forward (cell grouping) and reverse grouping (serum grouping) methods were used. The final blood group was confirmed only if both forward and reverse groups were identical. Donor’s age, sex, location of blood donation, dates of donation and blood groups with Rh factors were tabulated in register book.

### Analysis

Analysis of the data was performed using STATA version 13 [14] software. Two-sided Chi square tests for association were computed to detect differences in categorical variables (age groups, gender, and blood groups) with probability values (p-values) calculated at the 0.05 level of significance.

## Results

### Background characteristics of the blood donors

The donors had a mean age of 21 years (SD ± 5.7 years), were mainly male (73%) adults (donors 18 years and above). The distribution of ABO blood groups among the donors was 50.3, 25.0, 20.4 and 4.3% for O, A, B and AB blood groups respectively. The background characteristics of the donors are shown in Table 1.

**Table 1:** Background characteristics of the donors

Characteristic	Total n (%)	Male n (%)	Female n (%)	p-value
Age group				
<18 years (non adult)	6388 (27.2)	3206 (18.7)	1810 (28.3)	<0.001
≥18 years (adult)	17,116 (72.8)	13,910 (81.3)	4578 (71.7)	
ABO group				
A	5874 (25)	4253 (24.85)	1621 (25.40)	0.255
AB	998 (4.25)	706 (4.12)	292 (4.58)	
B	4791 (20.39)	3531 (20.63)	1260 (19.74)	
O	11,833 (50.36)	8624 (50.39)	3209 (50.28)	
Rhesus group				
RH (D) positive	23,027 (97.97)	16,728 (97.73)	6299 (98.61)	<0.001
RH (D) negative	477 (2.03)	388 (2.27)	89 (1.39)	

The proportion of non adult donors (<18 years) was significantly higher among the female than the male donors (p-value <0.001). A significantly higher proportion of males than females were Rhesus (D) negative (p-value <0.001).

### Distribution of Rhesus (D) factors among the ABO blood groups

The proportion of Rhesus (D) positive to Rhesus (D) negative blood donors was 97.97–2.03% respectively. The proportions of Rhesus (D) positive and negative blood donors were not significantly different among the ABO blood groups (p = 0.501). The distribution of Rhesus (D) factor among the different ABO blood group is shown in Table 2.

**Table 2:** Distribution of Rhesus (D) factors among the different ABO blood groups

Blood group	Rhesus (D) Positive n (%)	Rhesus (D) Negative n (%)	p-value
A	5767 (98.14)	109 (1.86)	0.501
AB	986 (98.21)	18 (1.79)	
B	4697 (98.04)	94 (1.96)	
O	11,577 (97.84)	256 (2.16)	

## Discussion

The findings of this study show that the blood group O occurs most frequently among the donors and blood group AB is the least common in southwestern Uganda. Knowledge of the distribution of ABO and Rhesus blood groups is an important element in determining the direction of recruitment of voluntary blood donors as required in each region and for effective management of blood banks inventory, be it at a facility of a small local transfusion service or regional or national transfusion services. It is therefore imperative to determine and have information on the distribution of ABO and Rhesus blood group systems of different ethnic groups in any population where blood transfusion services are being offered.

These findings are consistent with studies done elsewhere in Africa [15, 16]. A similar distribution of ABO blood groups have been seen in studies conducted among the Bangladesh population, Western Europeans, the African-American and Caucasian population of America [11, 17, 18] although the proportion of blood group O was slightly below 50%, ranging from 40 to 47%. Other studies in Australia by red cross society [6], and in USA by Mollison PL et al. [5, 28] have shown the commonest blood group was O, followed by A, B and AB, also consistent with our study findings. Unlike a study conducted elsewhere in Africa, this study did not determine the distribution of ABO and Rhesus group by ethnic group [19].

However, in contrast to our study, studies conducted in India [20] and Pakistan [21] showed blood group B was the most predominant, followed by blood group O, A and AB. While another study done in Nepal by Pramanik et al. [11] found the commonest blood group as group A, followed by blood group O, B and AB. In all the studies cited and including our study, blood group AB is the least distributed among the population of the world.

This study reveals that Rhesus (D) negativity has the lowest distribution among the donors which is similar to other studies conducted on other African continent. The identification of Rhesus blood system is important to prevent erythroblastosis fetalis, which commonly arises when a Rhesus negative mother carries Rhesus positive fetus. With the low incidence of Rhesus negativity in our setting the number of cases of haemolytic disease of the newborn (HDN) are expected to be much lower. Worlledge et al. [22] also found a low incidence of Rhesus negativity among the Nigerians, between 1.69 and 5.5%. Collectively these findings confirm low incidence of Rhesus (D) negative among the African population.

The frequency of Rh negativity, is less in Africans, Asians (mainly Chinese) and American blacks compared to Caucasians [23]. In most parts of India, the incidence of Rhesus (D) negative blood group varies from 2 to 6%. About 5–11% of donors all over the world are detected as Rhesus (D) negative except in Britain and USA, where the distribution of Rhesus (D) negative is 15 and 17% respectively [5, 28].

In this study there was no difference distribution of donors among the ABO blood groups by gender. However a higher proportion of Rhesus (D) negative was found among the male donors. It was also observed that males in southwestern Uganda constitute the majority group of blood donors. A similar trend has been observed elsewhere in Africa [24] and many parts of India [25, 26]. The dominance of male over female in blood donation exercise can be attributed to the fact that in an African context there is a general belief that men are healthier than women [24] and thus are more suitable for blood donation. Women in menstruating age group lose blood every month and are anemic, so unfit for blood donation and are eliminated by the pre-donation screening exercise. Other obstetrical factors including pregnancy and breastfeeding render them further from donating blood. In India social taboo, cultural habits, lack of motivation and fear of blood donation have been cited as some of the reasons why female donors are very few [27]. However for southwestern Uganda, these findings suggest that emphasis should now be placed on recruitment of female donors to improve supplies of Rhesus (D) positive blood.

In contrast to findings of studies elsewhere that demonstrate males as the most prevalent donors, our study results show that non-adult females in Uganda constitute the majority of donors. About 85% of Uganda’s voluntary non-remunerated blood donors are of school going age, to which the non-adult females belong because of the government’s policy on affirmative action and women emancipation. Further the pre-donation education given to potential donors in Uganda targets women encouraging them to donate willingly.

The sequence of ABO distribution among the rural population in southwestern Uganda is; O > A > B > AB. While the frequency of Rhesus (D) negative is very low in rural southwestern Ugandan and is mainly among males. Further, males are the predominant blood donors in the region. The blood bank services in southwestern Uganda need to develop innovative strategies targeting female donors. This is likely to boost stocks for the blood bank in the region and also minimize the collection of Rhesus (D) negative supplies. Similar studies should be undertaken for the other regions of the country to establish the blood group distribution. Collectively these studies would provide the national blood bank services with information critical for supply forecasting and blood inventory management.

## List of abbreviations

AB: Andrew Byamungu
CDC: centre for disease control and prevention
CI: confidence interval
EMM: Edgar Mugema Mulogo
FB: fred bagenda
FRC: faculty research committee
g/dL: gramme per deciliter
HDN: hemolytic disease of the new born
REC: research ethics committee
Kg: kilogram
mmHg: milliliter mercury
MUST: mbarara university of science and technology
Rh(D): rhesus (D) antigen/blood group
ROA: Richard Onyuthi Apecu
SD: standard deviation
UBTS: uganda blood transfusion services
USA: United States of America
